# Time to Pregnancy, Obstetrical and Neonatal Outcomes after Breast Cancer: A Study from the Maternity Network for Young Breast Cancer Patients

**DOI:** 10.3390/cancers13051070

**Published:** 2021-03-03

**Authors:** Julie Labrosse, Anne Lecourt, Alice Hours, Clara Sebbag, Aullene Toussaint, Enora Laas, Florence Coussy, Beatriz Grandal, Elise Dumas, Eric Daoud, Charlotte Morel, Jean-Guillaume Feron, Matthieu Faron, Jean-Yves Pierga, Fabien Reyal, Anne-Sophie Hamy

**Affiliations:** 1 Department of Surgery, Institut Curie, 75005 Paris, France; julie.labrosse@aphp.fr (J.L.); anne.lecourt@gmail.com (A.L.); alice.hours@aphp.fr (A.H.); clara.sebbag@curie.fr (C.S.); enora.laas@curie.fr (E.L.); charlotte.morel@aphp.fr (C.M.); jean-guillaume.feron@curie.fr (J.-G.F.); 2 Centre René Hughenin, Medical Oncology Department, 92210 Saint Cloud, France; aullene.toussaint@curie.fr (A.T.); florence.coussy@curie.fr (F.C.); anne-sophie.hamy-petit@curie.fr (A.-S.H.); 3 Residual Tumor & Response to Treatment Laboratory, RT2Lab, Translational Research Department, INSERM, U932 Immunity and Cancer, F-75005 Paris, France; beatriz.grandalrejo@curie.fr (B.G.); elise.dumas@curie.fr (E.D.); eric.daoud@curie.fr (E.D.); 4 Department of Biostatistics and Epidemiology, INSERM 1018 CESP Oncostat Team, Gustave, Roussy Cancer Campus, 94800 Villejuif, France; matthieu.faron@gustaveroussy.fr; 5 Medical Oncology Department, Institut Curie, 75005 Paris, France; jean-yves.pierga@curie.fr

**Keywords:** time to pregnancy, breast cancer, obstetrical outcomes, neonatal outcomes

## Abstract

**Simple Summary:**

Little is known on the time necessary to obtain a pregnancy after breast cancer and on the subsequent obstetrical and neonatal outcomes. We analyzed a cohort of breast cancer survivors aged 18–43 years old at diagnosis and having at least one pregnancy after cancer. While 71% of patients attempted to be pregnant, 18% of pregnancies were unplanned. Most pregnancies (86%) were obtained spontaneously and a minority occurred after assisted reproductive technologies (11%). We showed that median time to evolutive pregnancy was 5.6 months, and only menstrual cycles before pregnancy was significantly associated with time to pregnancy after multivariate analysis. Neonatal outcomes were similar to general population, and none of the prior BC treatments affected parameters of newborns. Our findings provide reassuring data for pregnancy counseling both in terms of delay and outcome. Our results also highlight the importance of appropriate contraceptive counseling after breast cancer to avoid unplanned pregnancies.

**Abstract:**

Although an increasing number of young breast cancer (BC) patients have a pregnancy desire after BC, the time necessary to obtain a pregnancy after treatment and subsequent outcomes remain unknown. We aimed to determine the time to evolutive pregnancy in a cohort of BC survivors and subsequent obstetrical and neonatal outcomes. We analyzed BC patients treated at Institut Curie from 2005–2017, aged 18–43 years old (y.o.) at diagnosis having at least one subsequent pregnancy. 133 patients were included, representing 197 pregnancies. Mean age at BC diagnosis was 32.8 y.o. and at pregnancy beginning was 36.8 y.o. 71% pregnancies were planned, 18% unplanned and 86% spontaneous. 64% pregnancies resulted in live birth (*n* = 131). Median time from BC diagnosis to pregnancy beginning was 48 months and was significantly associated with endocrine therapy (*p* < 0.001). Median time to pregnancy was 4.3 months. Median time to evolutive pregnancy 5.6 months. In multivariate analysis, menstrual cycles before pregnancy remained significantly associated with time to pregnancy and endocrine therapy with time evolutive to pregnancy. None of the BC treatments (chemotherapy/endocrine therapy/trastuzumab) was significantly associated with obstetrical nor neonatal outcomes, that seemed comparable to global population. Our findings provide reassuring data for pregnancy counseling both in terms of delay and outcome.

## 1. Introduction

Breast cancer (BC) is the most frequent cancer in women of childbearing age [1]. Among all female cancers diagnosed, up to 40% is BC by the age of 40 years old [2]. Due to substantial advances in treatment and care, the prognosis of young BC patients is constantly improving, and the 5-year survival rate of women aged from 15 to 44 years at BC diagnosis reaches 90% [3,4]. Besides, an increasing number of women postpone childbearing due to personal, educational or professional reasons [5,6]. The proportion of first births to women aged 35 years old or more is eight times higher than 30 years ago [7]. Hence, given better prognoses and the tendency to have children later in life, it is likely that an increasing number of young BC patients will have a pregnancy desire after BC [8]. Up to 50% of them declared wishing to conceive after treatment [1].

Since BC in women of reproductive age are known to be more aggressive and associated with an overall worse prognosis [9,10,11,12], these patients are more likely to receive systemic treatments such as chemotherapy that can transiently or permanently impair their gonadal function and fertility [13,14,15]. International guidelines recommend an early and prompt discussion to inform on the possible risks and available strategies to preserve fertility [16,17], notably since initiating a pregnancy after BC does not seem to affect prognosis [18,19,20,21]. On the contrary, some studies suggested that women becoming pregnant after BC had a more favorable prognosis than those with no pregnancy [22,23,24,25,26]. Indeed, it is possible that patients self-select to become pregnant when their prognosis is favorable, known as the “healthy mother effect” [27]. For BC patients, embryo and/or oocyte cryopreservation after controlled ovarian stimulation, in vitro maturation of oocytes and ovarian tissue cryopreservation can be considered [28], and GnRH agonists during chemotherapy also represent an option.

Altogether, data on fertility of BC patients after treatment are scarce. The overall pregnancy rate after BC varies from 3.6% to 16% [19,20,23,24,25,29,30,31], but these rates do not represent the fertility rate, as many patients may not wish to conceive after BC. Little is known on the time necessary to obtain a pregnancy after BC treatment. So far, studies have only described the time lapse between the end of treatments and pregnancy onset. The interval between the moment patients actually start their pregnancy attempt and the moment they become pregnant remains unknown. Furthermore, very few data exist on the obstetrical and neonatal outcomes of pregnancies obtained after BC. 

The aim of the present study was to determine the time to evolutive pregnancy, defined as time from first attempt to the occurrence of an evolutive pregnancy, in a cohort of BC survivors and to describe the subsequent obstetrical and neonatal outcomes.

## 2. Materials and Methods

### 2.1. Patients and Tumors

Our retrospective study included female patients with early BC and no prior history of cancer, treated at Institut Curie between 2005 and 2017, aged from 18 to 43 years old at BC diagnosis, and who had at least one pregnancy after BC diagnosis. Information on clinical characteristics (age, body mass index) and tumor characteristics (tumor size and grade, ER, PR, *HER2* status, lymph node involvement, number of mitosis, ki67) were retrieved from electronic medical records. Histological grade was described according to the Elston-Ellis modification of the Scarff-Bloom-Richardson grading system [32]. Hormone-receptor expression was analyzed by immunohistochemistry. Tumors were considered positive for estrogen receptor (ER) or progesterone receptor (PR) if 10% of carcinomatous cells displayed positive staining, as recommended by French guidelines [33]. *HER2* status was determined according to American Society of Clinical Oncology (ASCO) recommendations [34]. Based on immunohistochemistry surrogates, pathological breast cancer subtypes were defined as follows: tumors positive for either ER or PR and negative for *HER2* were classified as luminal; tumors positive for *HER2* were considered *HER2*-positive BC; tumors negative for ER, PR, and *HER2* were considered triple negative BC (TNBC). The study was approved by the Institut Curie Breast Cancer Study Group and was conducted according to institutional and ethical rules concerning research on patients. Written informed consent from patients was not required by French regulations.

### 2.2. Treatments

Patients were treated according to national guidelines. For patients receiving neoadjuvant chemotherapy, surgery was performed four to six weeks after the end of chemotherapy. Trastuzumab was used in an adjuvant and/or neoadjuvant setting for *HER2*-positive breast cancer according to national guidelines. Most patients received adjuvant radiotherapy. Endocrine therapy (tamoxifen, aromatase inhibitor, and/or GnRH agonists) was prescribed when indicated.

### 2.3. Identification of Pregnancy Cases

Pregnancy cases were identified by text mining technology as previously described [35]. Briefly, the text mining approach consisted in applying a keyword filter (“accouch *” or “enceinte”, French terms for “deliver *” and “pregnant”, respectively) on the medical electronic records of patients to select a subset of files. This text mining method has previously been described and has proven to be more efficient than manual curation of files. Files identified by text mining technique were then manually checked to confirm the presence of a pregnancy. 

### 2.4. Pregnancy Planning, Time to Evolutive Pregnancy, Obstetrical and Neonatal Outcomes

To determine pregnancy planning, time to evolutive pregnancy, obstetrical and neonatal outcomes, patients were contacted by telephone and were interrogated according to a predetermined questionnaire by two medical residents (AL, AH). Patients who had elective abortion, or abortion for medical reasons, and patients who had experienced relapse were not contacted for ethical reasons. The telephone call was repeated up to 3 times if patients were not reached. For patients that could not be reached by telephone after 3 attempts, electronic medical files were manually explored to retrieve data. Data were considered “very reliable” when obtained from the telephone conversation with the patient and were considered “fairly reliable” when manually retrieved in electronical medical records. Data were considered “not available” otherwise.

The pregnancy was considered planned if the patient mentionned orally a pregnancy desire and/or active attempt to conceive, or if such mention was found in the electronical health record or, was considered unplanned else.

Time to evolutive pregnancy was defined as the time from first attempt to get pregnant to the occurrence of pregnancy leading to a live birth (in months). Time to pregnancy was defined as a secondary endpoint and was defined as the time from first attempt to get pregnant to the occurrence of pregnancy, irrespective of the pregnancy outcome. By construction, as no pregnancy attempt could be considered for pregnancies that had not been planned, the time to pregnancy was set to “not available”. 

Obstetrical and neonatal outcomes included information on obtstetrical complications, delivery (term, delivery route), newborn parameters at birth (weight, size, head circumference, Apgar score at 5 min, gender) and lactation were recorded.

Small for gestational age (SGA) was defined by newborns having a weight below the 10th percentile for gestational age [36]. 

### 2.5. Statistical Analysis

The study population was described in terms of frequencies for qualitative variables, or medians and associated ranges for quantitative variables. To compare continuous variables among different groups, Wilcoxon-Mann-Whitney test was used for groups including less than 30 patients, and for variables displaying multimodal distributions, otherwise, we used student *t*-test. Association between categorical variables was assessed with the chi-square test, or with the Fisher’s exact test if at least one category included less than three patients. In boxplots, lower and upper bars represent the first and third quartile, respectively, the medium bar is the median, and whiskers extend to 1.5 times the inter-quartile range.

The relation between the time to pregnancy and the independent variables was assessed by a multiple linear model. In order to respect the hypothesis of normality of distribution of the residuals, the logarithm (base 10) of the time to pregnancy was used. Therefore the coefficient value can be interpreted as a multiplicative effect on the time (e.g., a coefficient of 1.5 means that for each more unit of the variable the time to pregnancy is multiplied by 10^1.5^). Prediction made by the model can then be back-transformed to the original time scale. Candidates variable for the multivariable analysis were selected by a Kruskal-Wallis test.

A forward stepwise selection procedure was used to establish the final multivariate model and the significance threshold was 5%. A significance threshold of 5% was used. 

Date of last update was censored to 29 March 2019. Analyses were performed with R software, version 3.1.2 (RStudio Team (2018). Rstudio: Integrated Development for R, RStudio, Inc., Boston, MA, USA, URL http://www.rstudio.com (accessed on 25 January 2021)).

## 3. Results

### 3.1. Patient Tumor Characteristics and Treatment

In all, a total of 133 patients who had at least one pregnancy after BC were included in the analysis (*n* = 197 pregnancies). Mean age of patients at BC diagnosis was 32.8 years old (y.o.) (Table 1). Tumors were mostly in T1 or T2 stage (41.5% and 36.8%, respectively), invasive (87.2%) and without nodal invasion (71.4%). BC subtypes repartition was as follows: luminal (*n* = 45, 40.9%), TNBC (*n* = 34, 30.9%) and *HER2*-positive (*n* = 31, 28.3%). All patients underwent surgery, 102 (76.7%) patients had chemotherapy, 53 had endocrine therapy (39.8%) and 30 (22.6%) had Trastuzumab 0.22 patients experienced relapse (local *n* = 13, regional *n* = 4, distant *n* = 5), and 1 patient died.

### 3.2. Pregnancies after BC

**Figure 1:**
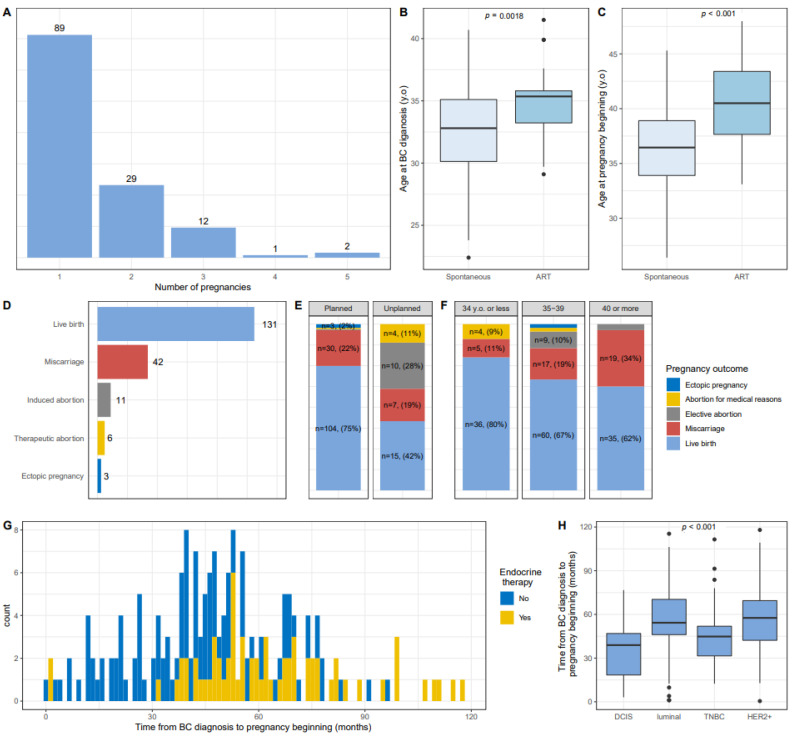
Characteristics of 197 pregnancies occuring after breast cancer in 133 patients. (**A**): Number of pregnancy by patient; (**B**): Age at BC diagnosis according to the spontaneous occurrence of the pregnancy or ART; (**C**): Age at pregnancy beginning according to the spontaneous occurrence of the pregnancy or ART; (**D**): Pregnancy outcomes; (**E**): Pregnancy outcomes by planned or unplanned nature of the pregnancy; (**F**): Pregnancy outcomes by age at pregnancy beginning; (**G**): Time from BC diagnosis to pregnancy beginning according to the use of endocrine therapy; (**H**): Time from BC diagnosis to pregnancy beginning by BC subtype. Effectives and percentages were removed from Figure 1E,F when absolute counts were 2 or below.

A total of 197 pregnancies were analyzed (Table S1). Mean age at first pregnancy was 36.8 years old (range 26.4–48.1 years). 40 patients had more than one pregnancy (two pregnancies, *n* = 29, three pregnancies, *n* = 12, four pregnancies (*n* = 1), five pregnancies (*n* = 2) (Figure 1A)). The pregnancy was planned in 71% cases (*n* = 139) and occurred while patients were not attempting to become pregnant in 18% of cases (*n* = 36) (missing information *n* = 22, 11%). Eight pregnancies occurred while the patient was under treatment (chemotherapy *n* = 4, radiotherapy *n* = 2, months following surgery (*n* = 2)). No factor was significantly associated with the occurrence of an unplanned pregnancy, except for treatment by endocrine therapy which was associated with a lower proportion of women experiencing unplanned pregnancy (Table S1).

A majority of pregnancies were spontaneous (86%), whereas 11% (*n* = 22) were obtained after ART (egg donation *n* = 15, ART without frozen material *n* = 4, ART with frozen material *n* = 3).

Among the 95 patients who planned a pregnancy, patients whose pregnancy occurred spontaneously (*n* = 78) were significantly younger than patients who had at least one pregnancy obtained after ART (*n* = 17) (age at BC diagnosis: 32.1 versus 34.6, *p* = 0.008, Figure 1B; age at first pregnancy after BC: 35.9 versus 39.8, *p* < 0.001, Figure 1C). Patients with spontaneous pregnancies were also less likely to receive endocrine therapy (*p* = 0.01) (Table S2).

Pregnancy outcomes were as follows: 64% of pregnancies (*n* = 131) resulted in live birth (*n* = 92 patients with one live birth; *n* = 18 patients with two live births; *n* = 1 patient with three live births); 21% (*n* = 42) resulted in miscarriages, 9% of patients had abortions (elective abortions *n* = 11 and abortions for medical reasons *n* = 6); 2% (*n* = 3) corresponded to ectopic pregnancies (Figure 1D).

Pregnancy outcomes were different according to whether the pregnancy had been planned or not (*p* < 0.001) (Figure 1E), with notably an abortion rate of 39% in case of unplanned pregnancy (elective abortions *n* = 10, abortion for medical reasons *n* = 4). Pregnancy outcome was also significantly associated with patients age at pregnancy beginning, with an increased risk of miscarriage with advancing age (*p* = 0.004, Figure 1F), but not according to whether the pregnancy was obtained after ART or spontaneously (Table S3).

### 3.3. Time to Pregnancy

#### 3.3.1. Time from Diagnosis to Pregnancy

Median time from BC diagnosis to the beginning of pregnancy was 48 months and was significantly associated with endocrine therapy (no endocrine therapy: 42 months versus endocrine therapy: 61 months, *p* < 0.001) and BC subtype (51.8 months for luminal tumors, 38.9 months for TNBC, 55.9 months for HER2-positive tumors and 37.4 months for in situ only tumors, respectively, *p* < 0.001).

#### 3.3.2. Time from First Attempt to the Occurrence of Pregnancy

**Figure 2:**
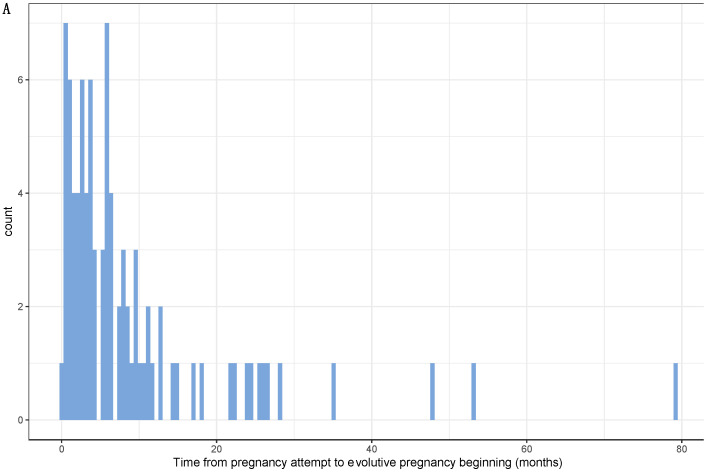

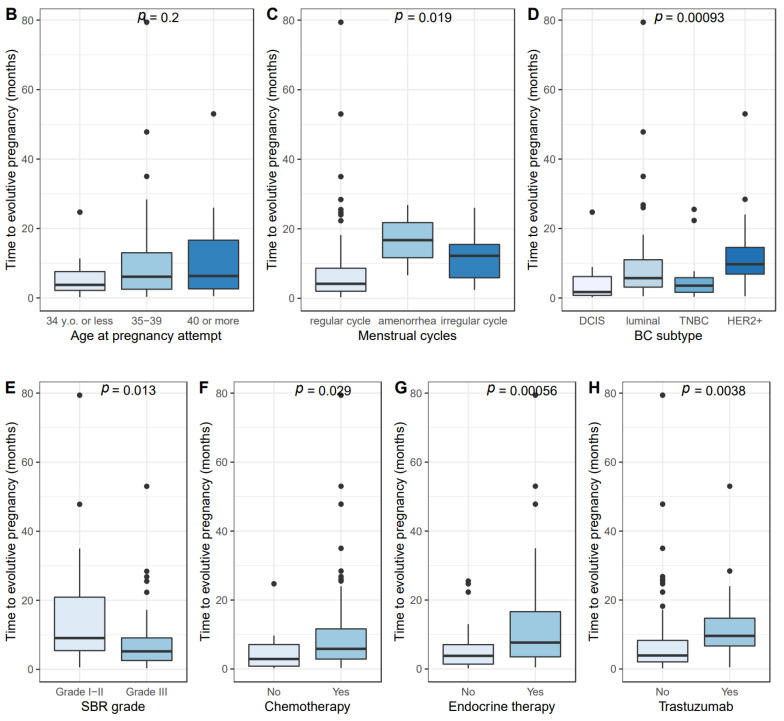
Time from pregnancy attempt to evolutive pregnancy begininng. (A). Histogram of distribution of time to evolutive pregnancy; Time to evolutive pregnancy according to age at pregnancy beginning (B), menstrual cycles before pregnancy attempt (C), BC subtype (D), tumor grade (E), previous chemotherapy (F), endocrine therapy (G), trastuzumab (H).

Out of 139 pregnancies where patients had attempted to be pregnant (*n* = 95), data on time from first attempt to the occurrence of first pregnancy was “very reliable” for 54 pregnancies (59%) and “fairly reliable” for 37 pregnancies (41%). Median time to achieve a pregnancy resulting in live birth was 5.6 months (Figure 2). After univariate analysis, time to evolutive pregnancy was significantly associated with BC subtype, tumor grade, chemotherapy, endocrine therapy, trastuzumab treatment, menstrual cycles before pregnancy attempt (Figure 2B–H) and only endocrine therapy remained significantly associated with time to evolutive pregnancy after multivariate analysis (Table 2).

Median time to pregnancy was 4.3 months (Figure S1). After univariate analysis, time to pregnancy was significantly associated with BC subtype, chemotherapy, endocrine therapy, menstrual cycles before pregnancy attempt (Supplemental Figure S1B–H), and only menstrual cycles before pregnancy remained significantly associated with time to pregnancy after multivariate analysis (Table S4).

When considering the subset of patients with “very reliable” information (*n* = 54), median time to obtain a pregnancy resulting in live birth was 3.7 months and median time to pregnancy was 3.6 months.

### 3.4. Obstetrical and Neonatal Outcomes

**Figure 3:**
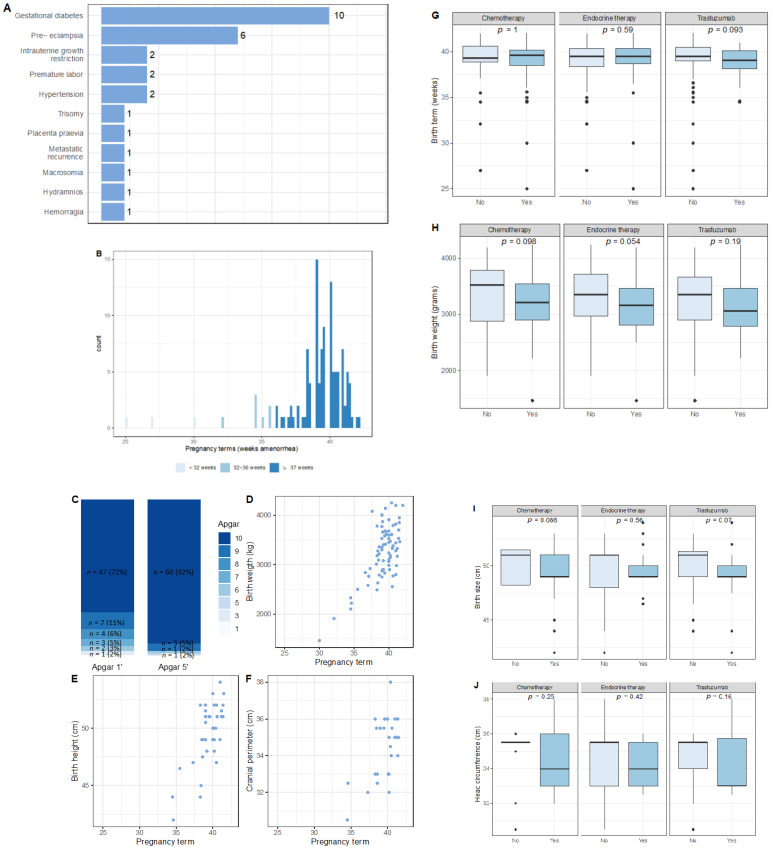
Obstetrical and neonatal outcomes on 131 evolutive pregnancies. (**A**): Obstetrical complications; (**B**): Histogram of delivery terms; (**C**): Apgar scores at 1 and 3 min (count and effectives); (**D**): Birth weight by delivery term; (**E**): Birth size by delivery term; (**F**): Cranial perimeter by delivery term; (**G**): Delivery term by previous cancer treatments; (**H**): Birth weight by previous cancer treatment; (**I**): Birth height by previous cancer treatments; (**J**): Head circumference (cm) by previous cancer treatments. Effectives and percentages were removed from Figure 3C when absolute counts were 2 or below.

Among 131 patients with an evolutive pregnancy, six patients had a twin pregnancy (Table 3). Pregnancy and obstetrical complications (*n* = 28) were as follows: gestational diabetes (*n* = 10), preeclampsia (*n* = 5), hypertension (*n* = 2), risk of preterm birth (*n* = 2), intrauterine growth restriction (*n* = 2), and various complications (*n* = 5) (Figure 3A).

Mean delivery term was 39.2 weeks, and eight babies (10.5%) were born preterm (<37 weeks of pregnancy) [moderate preterm (32–36 weeks of pregnancy), *n* = 7; very preterm (<32 weeks of pregnancy), *n* = 1] (Figure 3B). Labor was spontaneous for 44 patients (57.9%), induced for 23 patients (30.3%), and scheduled for nine patients (11.8). 53 patients had a vaginal delivery (62.4%) while 32 had a caesarean section (37.6%). 

Concerning neonatal outcomes, 1 and 5 min Apgar score were 10 in most of the newborns (72 and 92% respectively), mean birthweight was 3250 g, mean size at birth was 49.5 cm (range: 42–54 cm) and mean head circumference 34.5 cm (range: 30.5–36 cm) (Figure 3C–F). Neither birth terms, birth size, birth weight and head circumference were significantly associated with changes according to previous cancer treatments (Figure 3G–J). 

Data on both birth term and birth weight were available for 79 newborns, eight (10.1%) of which were SGA. The risk of SGA was not significantly associated to cancer treatments (Table S5).

Of the newborns 43were boys (46.7%) and 49 were girls (53.3%). One patient had a per-partum hemorrhage requiring embolisation and three patients had post-partum hypertensive disorders. Forty two newborns were breastfed (59.2%).

Neither late pregnancies (age at pregnancy beginning > 40 y.o., *n* = 39) (Table S6) nor pregnancies obtained after ART (Table S7) were significantly associated with obstetrical nor neonatal complications (except for a higher proportion of twin pregnancy with ART than without (17.6% vs. 2.7% respectively).

## 4. Discussion

In this cohort of young BC survivors with subsequent pregnancy, we found that: (i) a substantial proportion of pregnancies were unplanned; (ii) most patients became pregnant spontaneously at a mean age of 36.8 years old (ii); Median time to pregnancy resulting in a live birth was 5.6 months; (vi) subsequent obstetrical and neonatal outcomes were reassuring. 

Our study gives several insights into pregnancy-related issues in BC survivors of childbearing age:

1. 18% of pregnancies were unplanned, representing nearly one pregnancy out of five, and this rate notably includes eight pregnancies occurring during treatment. Of note, 39% of these unplanned pregnancies ended up with an abortion, whether elective or for medical reasons. Our results are consistent with previous literature, with abortion rates varying from 3% to 42% [1,19,20,23,24,25]. Contraception in breast cancer patient is a topic that has garnered little attention so far. However, it is particularly important because planning pregnancy in these patients is crucial from a medical point of view. For patients who do not wish to become pregnant, pregnancy should be actively avoided, particularly during chemotherapy and tamoxifen treatment, as these medications are known for their teratogenic effects. Our results highlight the fact that the contraception topic is insufficiently addressed in young breast cancer patients.
2. Most pregnancies occurred spontaneously, and the median time to a pregnancy resulting in a live birth was short. Our results are in line with the time to pregnancy reported in general population. A prospective cohort of 960 patients aged 30 to 44 years old found that median time to pregnancy was 3 months for women under 38 years old, 4 months for women aged 38-39 years old, 8 months for women aged 40–41 years old, and longer than 12 months for women aged 42 or older [37]. In a preconception cohort study, 46.3% women (*n* = 141) aged from 37 to 39 years old became pregnant within six cycles (95%CI [37.3–55.3]) [38]. In global population, time to pregnancy is known to increase with age and be longer in nulliparous women [37,38,39]. In a recent multicenter prospective cohort study, up to 69% of BC patients wishing to conceive became pregnant within 5 years after diagnosis. Younger age at diagnosis was significantly associated to the occurrence of a pregnancy [40]. However, to our knowledge, data on time from pregnancy attempt to pregnancy occurrence have not been published so far in a selected population of BC patients, and the results we present here are unprecedented. Previous studies mainly focused on the time interval between BC diagnosis and live birth, and provide delays varying from 23 to 45 months according to the different studies led so far [19,20,22,24,25,41,42,43,44,45,46]. In Gerstl et al.’s meta-analysis of 2 523 BC patients who became pregnant after BC treatment, the mean interval to the first pregnancy was 29 months (range: 11–63 months) and 40 months (range: 10–228 months) to a first live birth [46]. Consistently, the mean interval from surgery to first pregnancy was of 45.4 months in our cohort. We found that the time interval was significantly different according to HR status, which is consistent with the fact BC patients are generally advised to take endocrine therapy for at least 2 years before becoming pregnant [47].
3. Obstetrical and neonatal after breast cancer were reassuring. Our analyses report an overall miscarriage rate of 22%, and the miscarriage rate was significantly associated with increasing age. Gerst et al.’s meta-analysis [46] reported an overall early pregnancy loss rate of 12%, varying from 2% to 24% according to studies [1,19,20,23,24,25,42,44,45,48]. Age of patients is known to be a risk factor of miscarriage, with an increasing risk with age [49]. Our 22% miscarriage rate is to interpret in light with the fact that 29% of the patients were 40 y.o. and older at pregnancy beginning in our cohort.

In our cohort, we notified that the rate of both pre-eclampsia and gestational diabetes mellitus (3.8% and 7.6% of evolutive pregnancies, respectively) was not increased in women with prior BC concerning the general population [50,51]. These results are in line with a previous case-control study showing no excess risk of pre-eclampsia (3.1% vs. 1.2%, OR 2.54, 95% CI [0.49–13.32], *p* = 0.268) and gestational diabetes (7.9% vs. 5.5%, OR 1.48, 95% CI [0.61–3.57], *p* = 0.579) for 165 women with breast cancer before pregnancy compared with women without breast cancer [44].

The rate of caesarian sections in our study was 37.6%, which is in the range previously described in literature [43,52,53,54]. When compared to the rate of caesarian section in global population, some studies suggested that the risk might be increased in BC patients due to closer monitoring during pregnancy [53,54]. Concerning neonatal outcomes, 9% of babies were born preterm (<37 weeks of pregnancy), which is higher than in global population [55]. These results are in line with a review of 39 studies presented at the San Antonio Breast cancer symposium in December 2020, finding that the risk of preterm delivery (OR 1.45, 95% CI [1.11–1.88]) and low birth weight (OR 1.50, 95% CI [1.31–1.73]) were significantly higher for BC survivors compared to global population [56]. No significant increased risk of congenital abnormalities or other pregnancy or delivery complications were observed in our cohort. Consistently, a recent retrospective multicenter cohort study reported favorable fetal outcomes in 150 BC patients with deleterious germline BRCA mutations who gave birth after BC [57]. Moreover, 59.2% of the newborns of our cohort had breastfeeding, which is in line with the rate in global population (69%) [55]. 

Our study has several strengths. To our knowledge, it is the first to assess time from pregnancy attempt to pregnancy success in BC survivors, and this parameter represents a pragmatic endpoint as to help for pregnancy counselling. Indeed, studies led so far have only described the time interval from the end of treatments to pregnancy onset but not the real time to pregnancy i.e., the time interval from the moment patients actually start their pregnancy attempt to first pregnancy. Moreover, analyses were led on a large number of patients and on a wide range of data available. Limitations must be acknowledged. By construction, as the objectives of the study were to describe time to pregnancy and obstetrical and neonatal outcomes, our results are only applicable for women who conceived after BC and are irrelevant to women who did not have any pregnancy after BC. Therefore, our study does not provide data on infertility after BC, which remains particularly challenging to determine. Effective markers of fertility after BC are lacking. Notably, markers of ovarian reserve do not seem to be predictive of reproductive outcomes and pregnancies. Furthermore, although studies have reported overall pregnancy rates after BC, the missing data in most of them is whether patients were actually trying to get pregnant or not.

Our findings have clinical implications for BC survivors of reproductive age. Adequate contraceptive counselling should be provided as soon as BC is diagnosed and should be reevaluated throughout follow-up to ensure that patients are efficiently protected against unplanned pregnancies. For patients who wish to conceive, we provide reassuring data both in terms of delay to pregnancy and outcome. The time to spontaneously obtain a pregnancy after BC seems short. This point is crucial to deliver to patients in clinical practice because a significant proportion will interrupt their endocrine therapy for a pregnancy project and that this premature discontinuation might expose them to an increased risk of relapse. In women who consider this option, endocrine therapy should be resumed after delivery to complete the 5 to 10 years of treatment recommended [8]. In our study, nearly two thirds (62%) of patients had an evolutive pregnancy within the first 6 months after the first attempt to get pregnant. Our results also suggest that BC patients could be addressed to ART teams after this delay in order to investigate if infertility may explain failure to get pregnant. Additional prospective studies are required to validate independently our findings, and to provide long term safety follow-up data of children born after their mother had a breast cancer diagnosis. 

## Figures and Tables

**Table 1:** Patient and tumor characteristics (*n* = 133).

Variables	Characteristics	*n^1^*	%
Age at BC diagnosis (years)	32.8 [+/−4.0]		
Body Mass Index	22.1 [+/3.4]		
Subtype	Luminal	45	40.9
	TNBC	34	30.9
	*HER2*-positive	31	28.2
Clinical T stage (TNM) *	T0—Tis	18	13.5
	T1	55	41.5
	T2	49	36.8
	T3	9	6.8
	T4	2	1.5
Clinical N stage (TNM) *	N0	76	57.1
	N+	57	42.9
Invasive or DCIS	Invasive	116	87.2
	DCIS	17	12.8
Histological type	Non specific type (NST)	106	98.1
	Lobular	2	1.9
Grade	Grade I	4	3.7
	Grade II	31	29.0
	Grade III	72	67.3
Primary treatment	Surgery	88	66.2
	Neoadjuvant treatment	45	33.8
Type of surgery	Lumpectomy	60	57.1
	Mastectomy	45	42.9
Axillary surgery	Sentinel node biopsy (SNB)	21	20.2
	Axillary node dissection	78	75.0
	No axillary surgery	5	4.8
Lymph nodes involved	N-	95	71.4
	N+	38	28.6
Chemotherapy	Yes	102	76.7
	Anthracycline—taxanes	77	57.9
	Anthracycline	18	13.5
	Taxanes	7	5.3
	No	31	23.3
Radiotherapy	Yes	86	65
	No	47	35
Trastuzumab	Yes **	30	22.6
	No	103	77.4
Endocrine therapy	Yes	53	39.8
	No	80	60.2

Missing data: BMI, *n* = 28; BC subtype, *n* = 23; Histological type, *n* = 25; SBR grade, *n* = 26; BC surgery, *n* = 28; Axillar surgery, *n* = 29. ^1^ The “*n*” denotes the number of patients. In case of categorical variables, percentages are expressed. In case of continuous variables, mean value is reported, with standard deviation between brackets. * Clinical T stage (TNM) and clinical nodal status (TNM) refer to clinical presentation at BC diagnosis, i.e., prior to any treatment. ** 1 patient with an *HER2*-positive BC did not receive Trastuzumab.

**Table 2:** Factors associated to time to evolutive pregnancy in univariate and multivariate analysis.

Variable	Univariate Analysis						Multivariate Analysis		
	*n*	Mean	Median	Coefficient	CI	*p*	Coefficient	CI	*p*
Age at BC diagnosis (years)									
<34	63	8.3	5.2			0.64			
35–39	21	11.9	5.9	0.314	(−0.307–0.934)				
≥40	5	9.4	6.6	−0.109	(−1.253–1.036)				
Age at pregnancy attempt (years)									
<34	30	5.3	3.75			0.2			
35–39	45	11	6.1	0.397	(−0.178–0.973)				
≥40	14	12.3	6.3	0.562	(−0.228–1.352)				
Subtype									
Luminal	35	11.3	5.7			0.004			
TNBC	20	5.4	3.55	−0.62	(−1.219–0.021)				
HER2	19	13.4	9.7	0.392	(−0.217–1.001)				
Clinical T stage (TNM)									
T0-T1	49	9.6	5.2			0.83			
T2	35	8.8	6.1	0.126	(−0.422–0.674)				
T3-T4	5	8.9	3.8	0.008	(−1.154–1.17)				
Clinical N stage (TNM)									
N0	54	10.1	5.4			0.7			
N1-N2-N3	35	7.9	5.8	0.054	(−0.48–0.589)				
Invasive or DCIS									
Invasive	77	10	5.8			0.02			
DCIS	12	4.6	1.7	1.049	(0.316–1.781)				
Histological type									
Non specific type (NST)	71	6	0.624			0.02			
Lobular	2	41.1		0.963	(−0.615–2.541)				
Grade									
Grade I–II	22	15.9	9.05			0.01			
Grade III	51	7.9	5.2	−0.716	(−1.267–0.164)				
Lymph nodes involved									
N-	65	8.9	5.2			0.46			
N+	24	10.2	6.2	0.281	(−0.305–0.867)				
Chemotherapy									
Yes	70	10.4	5.85			0.03			
No	19	4.9	2.9	0.83	(0.217–1.443)				
Chemotherapy regimen									
Anthracycline—taxanes	51	10.2	5.9	0.902	(0.27–1.535)	0.1			
Anthracycline	15	12.7	3.9	0.864	(0.05–1.677)				
Taxanes	4	4.2	3.85	−0.212	(−1.507–1.083)				
Trastuzumab									
Yes	18	13.5	9.6			0.004			
No	71	8.2	3.9	0.792	(0.163–1.421)				
Endocrine therapy									
Yes	42	13.6	7.65			0.001			0.001
No	47	5.4	3.8	0.936	(0.451–1.421)		0.936	(0.451–1.421)	
Menstrual cycle before pregnancy attempt									
Regular Cycle	66	8.5	4.15			0.02			
amenorrhea	2	16.7	16.7						
Irregular cycle	12	12.1	12.2	1.136	(−0.463–2.736)				

Note: n denotes the effectives by class of each levels; mean and median represent the average and median values of time to evolutive pregnancy in each group and are presented for descriptive purpose; coefficients are the coefficient derived from the linear regression model with their corresponding confidence intervals and are calculated based on logged data.

**Table 3:** Obstetrical and neonatal outcomes on 131 evolutive pregnancies.

Variables	*n*^1^ (%)
Mutiple pregnancy	
No	125 (95.4)
Yes	6 (4.6)
Obstetrical complications	
Gestational diabetes	10 (38.5)
Pre-eclampsia	5 (19.2)
Hypertension	2 (7.7)
Premature labor	2 (7.7)
Intra-uterine growth restriction	2 (7.7)
Hemorragia	1 (3.8)
Hydramnios	1 (3.8)
Macrosomia	1 (3.8)
Metastatic recurrence	1 (3.8)
Placenta praevia	1 (3.8)
Metabolic and vascular complications	
No	114 (87.0)
Yes	17 (13.0)
Pregnancy term (weeks of amenorrhea)	39.2 [2.0]
<32 weeks	1 (0.9)
32–36 weeks	7 (6.1)
≥37 weeks	107 (93.0)
Labor	
Induction	23 (30.3)
Scheduled	9 (11.8)
Spontaneous	44 (57.9)
Delivery route	
Caesarean section	32 (37.6)
Vaginal delivery	53 (62.4)
Birth weight (in grams)	3253.0 [SD: 550.8]
Birth size (in centimeters)	49.5 [SD: 2.6]
Cranial perimeter (in centimeters)	34.5 [SD: 1.7]
Apgar	
1 min	9.3 [SD: 1.7]
5 min	9.8 [SD: 0.8]
Gender	
Female	49 (53.3)
Male	43 (46.7)
Post-partum complications	
Embolisation	1 (25.0)
Hypertension	3 (75.0)
Breastfeeding	
No	29 (40.8)
Yes	42 (59.2)

SD: standard deviation; 1 The “n” denotes the number of patients. In case of categorical variables, percentages are expressed between brackets. In case of continuous variables, mean value is reported, with standard deviation between brackets.

## Data Availability

The data presented in this study are available on request from the corresponding author.

## Supplementary Materials

The following are available online at https://www.mdpi.com/2072-6694/13/5/1070/s1, Figure S1: Time from pregnancy attempt to pregnancy begininng, Table S1: Patient characteristics according to planned or unplanned pregnancy (n = 197) pregnancies), Table S2: Patient characteristics according to the performance of ART after BC, Table S3: Pregnancy outcomes by pregnancy planning, spontaneous occurrence or need for ART, and age class at pregnancy beginning, Table S4: Univariate and multivariate analysis of patient and tumor characteristics with time from pregnancy attempt to pregnancy beginning, TableS5: Patient and tumor characteristics, by normal birthweight or small for gestational age (SGA) newborns, Table S6: Obstetrical and neonatal outcomes on 131 evolutive pregnancies, by age at pregnancy beginning, Table S7: Obstetrical and neonatal outcomes on 131 evolutive pregnancies, by spontaneous pregnancy or pregnancy obtained with ART.
