# Supplementary material for: Time to Pregnancy, Obstetrical and Neonatal Outcomes after Breast Cancer: A Study from the Maternity Network for Young Breast Cancer Patients
Source: Cancers (Basel). 2021 Mar 3;13(5):1070. doi: 10.3390/cancers13051070 (PMC7959151; doi:10.3390/cancers13051070)
Supplement: Supplementary file 1 [file cancers-13-01070-s001.pdf]

Supplementary Materials: Time to Pregnancy, Obstetrical and Neonatal Outcomes after Breast Cancer: A Study from the Maternity Network for Young Breast Cancer Patients

Julie Labrosse, Anne Lecourt, Alice Hours, Clara Sebbag, Aullene Toussaint, Enora Laas, Florence Coussy, Beatriz Grandal, Elise Dumas, Eric Daoud, Charlotte Morel, Jean-Guillaume Feron, Matthieu Faron, Jean-Yves Pierga, Fabien Reyal and Anne-Sophie Hamy

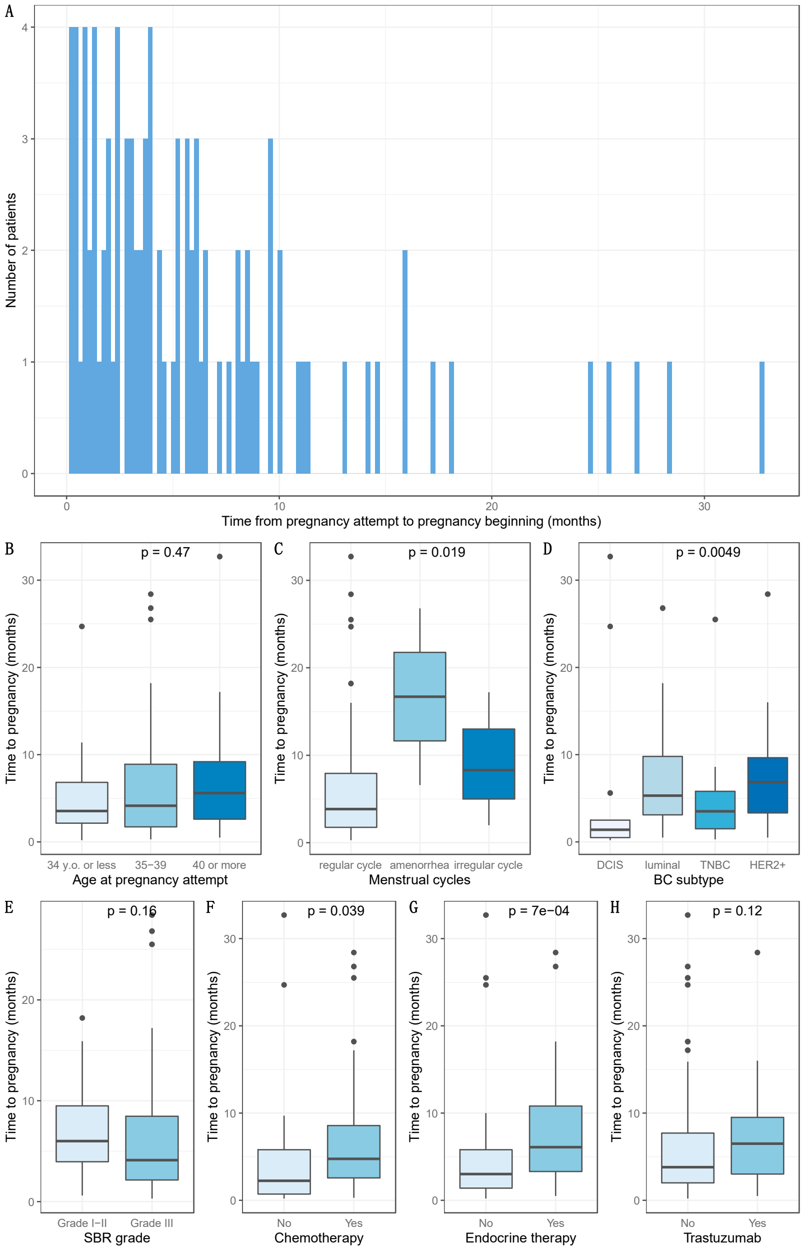

Figure S1. Time from pregnancy attempt to pregnancy begininng. (**A**). Histogram of distribution of time to pregnancy; Time to pregnancy according to age at pregnancy beginning (**B**), menstrual cycles before pregnancy attempt (**C**), BC subtype (**D**), tumor grade (**E**), previous chemotherapy (**F**), endocrine therapy (**G**), trastuzumab (**H**).

Table S1. Patient characteristics according to planned or unplanned pregnancy (*n* = 197) pregnancies).

Variables	Overall *n* = 197	Planned *n* = 139	Unplanned *n* = 36	*p*
Age at BC diagnosis (years)	32.8 [+/− 4.0]	32.7 [+/− 3.6]	33.7 [+/− 4.2]	0.17
Age at pregnancy beginning	36.8 [+/− 3.8]	37.2 [+/− 3.7]	36.2 [+/− 3.9]	0.16
Age at pregnancy beginning				0.53
34 y.o. or less	46 (23.8)	30 (21.9)	11 (30.6)	
35–39	91 (47.2)	65 (47.4)	16 (44.4)	
40 or more	56 (29.0)	42 (30.7)	9 (25.0)	
Subtype				0.08
Luminal	57 (37.3)	43 (39.8)	10 (34.5)	
TNBC	49 (32.0)	30 (27.8)	4 (48.3)	
HER2	47 (30.7)	35 (32.4)	5 (17.2)	
Clinical T stage (TNM)				0.92
Tis-T0-T1	110 (55.8)	76 (54.7)	21 (58.3)	
T2	72 (36.5)	55 (39.6)	13 (36.1)	
T3–T4	15 (7.6)	8 (5.8)	2 (5.6)	
Clinical N stage (TNM)				0.68
N0	122 (61.9)	85 (61.2)	24 (66.7)	
N1-N2-N3	75 (38.1)	54 (38.8)	12 (33.3)	
Invasive or DCIS				0.89
Invasive	164 (83.2)	116 (83.5)	31 (86.1)	
DCIS	33 (16.8)	23 (16.5)	5 (13.9)	
Histological type				0.88
Non specific type (NST)	149 (98.0)	105 (97.2)	27 (100.0)	
Lobular	3 (2.0)	3 (2.8)	0 (0.0)	
Grade				0.75
Grade I-II	55 (36.9	38 (35.2)	11 (40.7)	
Grade III	94 (63.1)	70 (64.8)	16 (59.3)	
Primary treatment				0.69
Surgery	135 (68.5)	96 (69.1)	23 (63.9)	
Neoadjuvant treatment	62 (31.5)	43 (30.9)	13 (36.1)	
Type of surgery				0.72
Lumpectomy	92 (56.1)	63 (54.8)	17 (60.7)	
Mastectomy	72 (43.9)	52 (45.2)	11 (39.3)	
Axillary surgery				0.90
Sentinel node biopsy (SNB)	42 (25.8)	29 (25.2)	7 (25.9)	
Axillary node dissection (AND)	111 (68.1)	80 (69.6)	18 (66.7)	
No axillary surgery	10 (6.1)	6 (5.2)	2 (7.4)	
Lymph nodes involved				0.99
N-	146 (74.1)	103 (74.1)	26 (72.2)	
N+	51 (25.9)	36 (25.9)	10 (27.8)	
Chemotherapy				0.66
Yes	140 (71.1)	104 (74.8)	25 (69.4)	
Anthracycline - taxanes	106 (53.8)	79 (56.8)	21 (58.3)	
Anthracycline	25 (12.7)	20 (14.4)	2 (5.6)	
Taxanes	9 (4.6)	5 (3.6)	2 (5.6)	
No	57 (28.9)	35 (25.2)	11 (30.6)	
Trastuzumab				0.49
Yes	44 (22.3)	33 (23.7)	6 (16.7)	
No	153 (77.7)	106 (76.3)	30 (83.3)	
Endocrine therapy				0.03
Yes	71 (36.0)	61 (43.9)	8 (22.2)	
No	126 (64.0)	78 (56.1)	28 (77.8)	

Missing data: Age at pregnancy beginning, *n* = 4; BC subtype, *n* = 44; BC subtype, *n* = 11; Histological type, *n* = 45; SBR grade, *n* = 48; BC surgery, *n* = 33; Axillar surgery, *n* = 34; [1] The "*n*" denotes the number of patients. In case of categorical variables, percentages are expressed between brackets. In case of continuous variables, mean value is reported, with standard deviation between brackets.

Table S2. Patient characteristics according to the performance of ART after BC.

Variables	No ART *n* = 78 (%)	One or more ART *n* = 17 (%)	*p*
Age at BC diagnosis	32.1 [3.6]	34.6 [3.3]	0.008
Age at first pregnancy	35.9 [3.2]	39.8 [3.8]	<0.001
Subtype			0.49
Luminal	28 (44.4)	6 (40.0)	
TNBC	19 (30.2)	3 (20.0)	
HER2	16 (25.4)	6 (40.0)	
Clinical T stage (TNM)			0.36
Tis- T0-T1	41 (52.6)	11 (64.7)	
T2	32 (41.0)	4 (23.5)	
T3-T4	5 (6.4)	2 (11.8)	
Clinical N stage (TNM)			0.41
N0	48 (61.5)	8 (47.1)	
N1-N2-N3	30 (38.5)	9 (52.9)	
Invasive or DCIS			0.52
Invasive	66 (84.6)	16 (94.1)	
DCIS	12 (15.4)	1 (5.9)	
Histological type			0.87
Non specific type (NST)	61 (98.4)	15 (93.8)	
Lobular	1 (1.6)	1 (6.2)	
Grade			0.96
Grade I-II	18 (28.6)	5 (33.3)	
Grade III	45 (71.4)	10 (66.7)	
Primary treatment			0.90
Surgery	51 (65.4)	12 (70.6)	
Neoadjuvant treatment	27 (34.6)	5 (29.4)	
Type of surgery			0.43
Lumpectomy	35 (54.7)	8 (72.7)	
Mastectomy	29 (45.3)	3 (27.3)	
Axillary surgery			0.50
Sentinel node biopsy (SNB)	15 (23.4)	1 (9.1)	
Axillary node dissection (AND)	46 (71.9)	9 (81.8)	
No axillary surgery	3 (4.7)	1 (9.1)	
Lymph nodes involved			0.69
N-	57 (73.1)	11 (64.7)	
N+	21 (26.9)	6 (35.3)	
Chemotherapy			0.48
Yes	60 (76.9)	15 (88.2)	
No	18 (23.1)	2 (11.8)	
Chemotherapy regimen			0.22
Anthracycline - taxanes	45 (57.7)	10 (58.8)	
Anthracycline	10 (12.8)	5 (29.4)	
Taxanes	5 (6.4)	0 (0.0)	
Trastuzumab			0.63
Yes	16 (20.5)	5 (29.4)	
No	62 (79.5)	12 (70.6)	
Endocrine therapy			0.01
Yes	31 (39.7)	13 (76.5)	
No	47 (60.3)	4 (23.5)	

Table S3. Pregnancy outcomes by pregnancy planning, spontaneous occurrence or need for ART, and age class at pregnancy beginning.

Variables		Live Birth	Miscar-riage	Elective Abortion	Abortion for Medical Reasons	Ectopic Preg-nancy	*p*
		N = 131	*N* = 42	*N* = 11	*N* = 6	*N* = 3	
Pregnancy planning	attempt	104 (87.4)	30 (81.1)	1 (9.1)	1 (20.0)	3 (100.0)	<0.001
	No attempt	15 (12.6)	7 (18.9)	10 (90.9)	4 (80.0)	0 (0.0)	

Pregnancy occurrence	Spontaneous	111 (86.7)	37 (88.1)	11 (100.0)	6 (100.0)	3 (100.0)	0.56
	ART	17 (13.3)	5 (11.9)	0 (0.0)	0 (0.0)	0 (0.0)	
Age at pregnancy beginning	≤34 years old	36 (27.5)	5 (12.2)	0 (0.0)	4 (66.7)	0 (0.0)	0.004
	35–39 years old	60 (45.8)	17 (41.5)	9 (81.8)	2 (33.3)	2 (100.0)	
	≥40 years old	35 (26.7)	19 (46.3)	2 (18.2)	0 (0.0)	0 (0.0)	

Table S4. Univariate and multivariate analysis of patient and tumor characteristics with time from pregnancy attempt to pregnancy beginning.

Variable	Univariate Analysis						Multivariate analysis		
	n	Mean	Median	Coeff.	CI	*p*	Coeff	CI	*p*
Age at BC diagnosis (years)									
< 34	63	5.9	3.9			0.59			
35-39	26	7.8	5.4	0.232	(−0.287–0.751)				
≥ 40	5	4.2	5	-0.35	(−1.385–0.685)				
Age at pregnancy attempt (years)									
< 34	30	5.1	3.6			0.47			
35-39	46	6.6	4.15	0.056	(−0.466–0.579)				
≥ 40	18	8	5.6	0.387	(−0.277–1.051)				
Subtype									
Luminal	35	7.2	5.3			0.04			
TNBC	21	4.6	3.5	-0.531	(−1.044–−0.017)				
HER2	22	7.7	6.9	0.034	(−0.472-0.541)				
ClinicalTstage(TNM)									
Tis-T0-T1	52	6.6	4			0.73			
T2	35	5.7	4.3	0.125	(−0.364–0.614)				
T3-T4	7	8.2	4.5	0.336	(−0.564–1.236)				
ClinicalNstage(TNM)						0.54			
N0	55	6.6	3.9						
N1-N2-N3	39	6	5	0.112	(−0.355–0.579)				
InvasiveorDCIS									
Invasive	81	6.5	5			0.008			
DCIS	13	5.7	1.4	0.968	(0.331–1.605)				
Grade									
GradeI-II	23	7.1	6			0.15			
GradeIII	54	6.4	4.1	-0.315	(−0.786–0.156)				
Lymphnodesinvolvedafterhistologicalexamination									
N-	68	6.2	3.9			0.2			
N+	26	6.8	5.9	0.354	(−0.156–0.863)				
Chemotherapy									
Yes	74	6.6	4.8			0.04			
No	20	5.6	2.3	0.688	(0.143–1.233)				
Chemotherapyregimen									
Anthracycline-taxanes	54	6.7	5.2	0.733	(0.166–1.3)	0.17			
Anthracycline	15	6.9	3.9	0.734	(−0.005–1.474)				
Taxanes	5	4.2	4.5	0.061	(−1.022–1.143)				
Trastuzumab									
Yes	21	7.5	6.5			0.12			
No	73	6.1	3.8	0.76	(0.327–1.194)				
Endocrinetherapy									
Yes	45	7.9	6.1			0.001			
No	49	4.9	3	0.385	(−0.162–0.932)				
Menstrualcyclebeforepregnancyattempt									
RegularCycle	70	5.9	3.9			0.019			0.019
Irregularcycle	13	8.7	8.3	1.333	(−0.129–2.795)		1.333	(−0.129–2.795)	

Amenorrhea	2	16.7	16.7	0.713	(0.097–1.329)	0.713 (0.097–1.329)

Note : n denotes the effectives by class of each levels; mean and median represent the average and median values of time to pregnancy in each group, and are presented for descriptive purpose ; coeff. are the coefficient derived from the linear regression model with their corresponding confidence intervals, and are calculated based on logged data.

Table S5. Patient and tumor characteristics, by normal birthweight or small for gestational age (SGA) newborns.

**Variables**	**No SGA** *n* = 71 (%)	**SGA** *n* = 8 (%)	*p*
Age at BC diagnosis	32.6 [3.9]	33.4 [3.2]	0.56
Subtype			0.80
Luminal	31 (55.4)	3 (42.9)	
TNBC	14 (25.0)	2 (28.6)	
HER2	11 (19.6)	2 (28.6)	
Clinical T stage (TNM)			0.71
T0-Tis	9 (12.7)	0 (0.0)	
T1	33 (46.5)	4 (50.0)	
T2	24 (33.8)	4 (50.0)	
T3	4 (5.6)	0 (0.0)	
T4	1 (1.4)	0 (0.0)	
Clinical N stage (TNM)			1.00
N0	50 (70.4)	6 (75.0)	
N1-N2-N3	21 (29.6)	2 (25.0)	
Invasive or DCIS			0.51
Invasive	60 (84.5)	8 (100.0)	
DCIS	11 (15.5)	0 (0.0)	
Histological type			1.00
Non specific type (NST)	56 (96.6)	8 (100.0)	
Lobular	2 (3.4)	0 (0.0)	
Grade			0.58
Grade I	2 (3.6)	0 (0.0)	
Grade II	18 (32.7)	4 (50.0)	
Grade III	35 (63.6)	4 (50.0)	
Primary treatment			0.27
Surgery	54 (76.1)	8 (100.0)	
Neoadjuvant treatment	17 (23.9)	0 (0.0)	
Type of surgery			0.53
Lumpectomy	36 (56.2)	3 (37.5)	
Mastectomy	28 (43.8)	5 (62.5)	
Axillary surgery			0.61
Sentinel node biopsy (SNB)	16 (25.4)	1 (12.5)	
Axillary node dissection (AND)	45 (71.4)	7 (87.5)	
No axillary surgery	2 (3.2)	0 (0.0)	
Lymph nodes involved			0.33
N-	52 (73.2)	4 (50.0)	
N+	19 (26.8)	4 (50.0)	
Chemotherapy			0.45
Yes	48 (67.6)	7 (87.5)	
No	23 (32.4)	1 (12.5)	
Chemotherapy regimen			0.39
Anthracycline - taxanes	31 (43.7)	6 (75.0)	
Anthracycline	13 (18.3)	1 (12.5)	
Taxanes	4 (5.6)	0 (0.0)	
Trastuzumab			0.85
Yes	11 (15.5)	2 (25.0)	
No	60 (84.5)	6 (75.0)	
Endocrine therapy			0.34
Yes	27 (38.0)	5 (62.5)	
No	44 (62.0)	3 (37.5)	

Table S6. Obstetrical and neonatal outcomes on 131 evolutive pregnancies, by age at pregnancy beginning.

Variables	Parameters	≤40 years old	≥40 years old	*p*
		N **= 96**	***N*** **= 35**	
Multiple pregnancy	No	93 (96.9)	32 (91.4)	0.4
	Yes	3 (3.1)	3 (8.6)	
Obstetrical complications	Gestational diabetes	6 (35.3)	4 (44.4)	0.55
	Pre-eclampsia	2 (11.8)	3 (33.3)	
	Hypertension	2 (11.8)	0 (0.0)	
	Premature labor	1 (5.9)	1 (11.1)	
	Intrauterine growth restriction	2 (11.8)	0 (0.0)	
	Hemorragia	0 (0.0)	1 (11.1)	
	Hydramnios	1 (5.9)	0 (0.0)	
	Macrosomia	1 (5.9)	0 (0.0)	
	Metastatic recurrence	1 (5.9)	0 (0.0)	
	Placenta praevia	1 (5.9)	0 (0.0)	
Obstetrical complications (metabolic and vascular)	No	86 (89.6)	28 (80.0)	0.25
	Yes	10 (10.4)	7 (20.0)	
Pregnancy term (weeks of amenorrhea)		39.4 (1.9)	38.6 (2.1)	0.07
Pregnancy term (weeks of amenorrhea)	< 32 weeks	1 (1.2)	0 (0.0)	0.52
	32-36 weeks	4 (4.8)	3 (9.7)	
	>= 37 weeks	79 (94.0)	28 (90.3)	
Labor	Induction	13 (24.1)	10 (45.5)	0.18
	Scheduled	7 (13.0)	2 (9.1)	
	Spontaneous	34 (63.0)	10 (45.5)	
Delivery route	Caesarean section	22 (36.1)	10 (41.7)	0.82
	Vaginal delivery	39 (63.9)	14 (58.3)	
Birth weight (in grams)		3295.9 (538.0)	3141.8 (580.3)	0.27
Birth size (in centimeters)		50.1 (2.4)	47.4 (2.1)	<0.01
Cranial perimeter (in centimeters)		34.8 (1.4)	33.3 (2.2)	0.06
Apgar 1 minute		9.2 (1.8)	9.5 (1.2)	0.44
Apgar 5 minutes		9.8 (0.9)	9.9 (0.2)	0.46
Gender	Female	37 (55.2)	12 (48.0)	0.7
	Male	30 (44.8)	13 (52.0)	
Post partum complications	Embolisation	0 (0.0)	1 (33.3)	1
	Hypertension	1 (100.0)	2 (66.7)	
Breastfeeding	No	25 (47.2)	4 (22.2)	0.11
	Yes	28 (52.8)	14 (77.8)	

Table S7. Obstetrical and neonatal outcomes on 131 evolutive pregnancies, by spontaneous pregnancy or pregnancy obtained with ART.

Variables	Parameters	Spontaneous	ART	*p*
		N **= 111**	***N*** **= 17**	
Multiple pregnancy	No	108 (97.3)	14 (82.4)	0.04
	Yes	3 (2.7)	3 (17.6)	
Obstetrical complications	Gestational diabetes	10 (45.5)	0 (0.0)	0.05
	Pre-eclampsia	2 (9.1)	3 (75.0)	
	Hypertension	2 (9.1)	0 (0.0)	
	Premature labor	2 (9.1)	0 (0.0)	
	Intrauterine growth restriction	2 (9.1)	0 (0.0)	
	Hemorragia	1 (4.5)	0 (0.0)	
	Hydramnios	1 (4.5)	0 (0.0)	
	Macrosomia	0 (0.0)	1 (25.0)	
	Metastatic recurrence	1 (4.5)	0 (0.0)	
	Placenta praevia	1 (4.5)	0 (0.0)	

Obstetrical complications (metabolic and vascular)	No	97 (87.4)	14 (82.4)	0.85
	Yes	14 (12.6)	3 (17.6)	
Pregnancy term (weeks of amenorrhea)		39.2 (2.0)	38.9 (1.5)	0.52
Pregnancy term (weeks of amenorrhea)	< 32 weeks	1 (1.0)	0 (0.0)	0.49
	32-36 weeks	7 (7.3)	0 (0.0)	
	>= 37 weeks	88 (91.7)	16 (100.0)	
Labor	Induction	17 (25.8)	6 (60.0)	0.07
	Scheduled	9 (13.6)	0 (0.0)	
	Spontaneous	40 (60.6)	4 (40.0)	
Delivery route	Caesarean section	27 (37.0)	5 (41.7)	1.00
	Vaginal delivery	46 (63.0)	7 (58.3)	
Birth weight (in grams)		3226.9 (561.4)	3433.0 (454.4)	0.27
Birth size (in centimeters)		49.5 (2.7)	49.2 (2.1)	0.84
Cranial perimeter (in centimeters)		34.4 (1.6)	34.7 (2.3)	0.83
Apgar 1 minute		9.2 (1.7)	9.9 (0.4)	0.32
Apgar 5 minutes		9.8 (0.8)	9.9 (0.4)	0.89
Gender	Female	46 (57.5)	3 (27.3)	0.12
	Male	34 (42.5)	8 (72.7)	
Post partum complications	Embolisation	1 (33.3)	0 (0.0)	1.00
	Hypertension	2 (66.7)	1 (100.0)	
Breastfeeding	No	25 (41.0)	4 (40.0)	1.00
	Yes	36 (59.0)	6 (60.0)	
